# A Mathematical Exploration of the Effects of Ischemia-Reperfusion Injury After a Myocardial Infarction

**DOI:** 10.3390/bioengineering12020177

**Published:** 2025-02-13

**Authors:** Mehtap Lafci Büyükkahraman, Houjia Chen, Benito M. Chen-Charpentier, Jun Liao, Hristo V. Kojouharov

**Affiliations:** 1 Department of Mathematics, Uşak University, Uşak 64200, Türkiye; 2 Department of Bioengineering, The University of Texas at Arlington, Arlington, TX 76010-0138, USA; hxc6348@mavs.uta.edu (H.C.); jun.liao@uta.edu (J.L.); 3 Department of Mathematics, The University of Texas at Arlington, Arlington, TX 76019-0408, USA; bmchen@uta.edu (B.M.C.-C.); hristo@uta.edu (H.V.K.)

**Keywords:** myocardial infarction, cardiomyocytes, neutrophils, macrophages, immune system, cytokines, *γδT* cells, stem cells, reperfusion injury, reactive oxygen species

## Abstract

Introduction: After myocardial infarction (MI), the heart undergoes necrosis, inflammation, scar formation, and remodeling. While restoring blood flow is crucial, it can cause ischemia-reperfusion (IR) injury, driven by reactive oxygen species (ROSs), which exacerbate cell death and tissue damage. This study introduces a mathematical model capturing key post-MI dynamics, including inflammatory responses, IR injury, cardiac remodeling, and stem cell therapy. The model uses nonlinear ordinary differential equations to simulate these processes under varying conditions, offering a predictive tool to understand MI pathophysiology better and optimize treatments. Methods: After myocardial infarction (MI), left ventricular remodeling progresses through three distinct yet interconnected phases. The first phase captures the immediate dynamics following MI, prior to any medical intervention. This stage is mathematically modeled using the system of ordinary differential equations: The second and third stages of the remodeling process account for the system dynamics of medical treatments, including oxygen restoration and subsequent stem cell injection at the injury site. Results: We simulate heart tissue and immune cell dynamics over 30 days for mild and severe MI using the novel mathematical model under medical treatment. The treatment involves no intervention until 2 h post-MI, followed by oxygen restoration and stem cell injection at day 7, which is shown experimentallyand numerically to be optimal. The simulation incorporates a baseline ROS threshold ($R_{c}$) where subcritical ROS levels do not cause cell damage. Conclusion: This study presents a novel mathematical model that extends a previously published framework by incorporating three clinically relevant parameters: oxygen restoration rate (ω), patient risk factors (γ), and neutrophil recruitment profile (δ). The model accounts for post-MI inflammatory dynamics, ROS-mediated ischemia-reperfusion (IR) injury, cardiac remodeling, and stem cell therapy. The model’s sensitivity highlights critical clinical insights: while oxygen restoration is vital, excessive rates may exacerbate ROS-driven IR injury. Additionally, heightened patient risk factors (e.g., smoking, obesity) and immunodeficiency significantly impact tissue damage and recovery. This predictive tool offers valuable insights into MI pathology and aids in optimizing treatment strategies to mitigate IR injury and improve post-MI outcomes.

## 1. Introduction

Coronary artery disease (CAD) is the leading cause of death in the United States and a significant global health challenge, contributing to 17.8 million deaths annually [1]. Myocardial infarction (MI), a severe manifestation of CAD, is often exacerbated by ischemia-reperfusion (IR) injury. During IR injury, reactive oxygen species (ROSs) generation increases, causing cellular damage and inflammation [2]. For acute coronary syndrome patients with hypoxemia (oxygen saturations less than 90%), oxygen supplementation is advised. Since there are no clinical benefits linked to oxygen supplementation in patients who are not hypoxic (oxygen saturations greater than 90%), it is not advised [3]. Although oxygen therapy and stem cell treatments offer potential benefits, challenges such as oxidative stress, reduced survival of transplanted cells, and adverse immune responses hinder recovery [4,5]. Recent works have been conducted by Jin et al. [6], Wang et al. [7], Voropaeva et al. [8], and Lafci Büyükkahraman et al. [9] on the mathematical modeling of the left ventricular (LV) remodeling process without considering medical treatments. These models included cardiomyocytes, macrophages, fibroblasts, transforming growth factor beta (TGF–β1), matrix metalloproteinase-9 (MMP–9), and collagen, as well as unactivated macrophages, classically (M1) and alternatively (M2) activated macrophages, tumor necrosis factor alpha (TNF–α), interleukin 1 (IL–1) and interleukin 10 (IL–10) cytokines for macrophages activation post-MI. Our recently published model [10] explicitly incorporates the post-MI regeneration process of cardiomyocytes under oxygen restoration and stem cell therapeutic medical intervention efforts. However, none of these models deal with the ischemia-reperfusion injury after myocardial infarction. They do not consider γδT cells and ROSs, which are crucial factors in IR. Recent discovery suggests that the main cytokine of the γδT-mediated immune response, IL–17A, may offer protection from IR injury [11]. This could have therapeutic significance since it has important immunological ramifications that could help create suitable stem cell treatments for IR injury after myocardial infarction [12]. This study introduces a novel mathematical model using nonlinear ordinary differential equations (ODEs) to analyze the post-MI regeneration process. The model incorporates the effects of ROSs, γδT cells, IL–17, oxygen restoration, and stem cell therapy. It generalizes prior work [10,13] to better capture the complex dynamics of IR injury and its impact on LV remodeling. This paper is structured as follows: Section 2.1 discusses the biological processes and modeling assumptions. Section 2.2 presents a new mathematical framework. Numerical simulations exploring the effects of IR injury under varying conditions are detailed in Section 3. Finally, Section 4 and Section 5 offer an analysis of findings and propose strategies for improving post-MI treatment outcomes.

## 2. Materials and Methods

### 2.1. Modeling Assumptions

Myocardial infarction (MI) results from persistent ischemia caused by a blocked coronary artery, leading to reduced oxygen supply to downstream myocardium [14,15], with oxygen levels in infarct hearts decreasing by up to 90% compared to uninjured hearts [16]. In response, left ventricular (LV) remodeling occurs, characterized by structural and functional changes in the LV, including alterations in size, shape, and function [6,17]. This complex process involves spatio-temporal interactions among various biological components, many of which are poorly understood due to limited experimental data and biological complexity [6]. The model assumes that cardiomyocytes, constituting about 30% of total cell volume in mouse ventricles [18], undergo significant apoptosis and necrosis following MI, with necrosis peaking between 12 h and 4 days [19]. Inflammatory and reparative responses are driven by immune cells, including neutrophils, monocytes, macrophages, and fibroblasts, as well as cytokines like IL–1, IL–10, and IL–17 [17,20]. Cellular recruitment, differentiation, and cytokine interactions are modeled to capture the dynamic changes in cell populations and extracellular matrix (ECM) composition during the recovery process. Cardiomyocyte death initiates inflammatory responses, with peak recruitment of neutrophils, monocytes, and macrophages occurring at defined time points post-MI [17,21]. IL–1 promotes M1 macrophage activation, while IL–10 drives M2 macrophage activation and fibroblast recruitment. IL–10 also inhibits IL–1 and self-regulates [7]. Fibroblasts are the primary source of collagen deposition, peaking around a week post-MI, and contribute to ECM remodeling and tensile strength improvement in infarct tissue [17,22]. M1 macrophages may transition to M2 macrophages at a constant rate, supporting tissue repair and regeneration [7]. The ECM facilitates cell migration, adhesion, and signaling, with collagen I and III playing critical roles in scar tissue formation and ventricular wall stiffening [19,23]. This model prioritizes the temporal resolution of cell population changes and cytokine interactions to simulate post-MI cardiac recovery.

### 2.2. Model Formulation

After myocardial infarction, left ventricular remodeling occurs in three distinct but related phases. The dynamics of the system immediately following MI, before the onset of any medical interventions, are taken into account in the first stage. It is mathematically modeled by the following system of ordinary differential equations:

(1) $$\begin{array}{ccc} \frac{dM_{c}}{dt} & = & -k_{15}M_{c}\frac{IL_{17}}{IL_{17}+c_{IL_{17}M_{c}}}-k_{16}NM_{c}-\mu_{M_{c}}M_{c}, \end{array}$$

(2) $$\begin{array}{ccc} \frac{dM_{d}}{dt} & = & k_{15}M_{c}\frac{IL_{17}}{IL_{17}+c_{IL_{17}M_{c}}}+\mu_{M_{c}}M_{c}-k_{2}M_{1}M_{d}-k_{17}NM_{d}, \end{array}$$

(3) $$\begin{array}{ccc} \frac{dIL_{1}}{dt} & = & k_{3}M_{d}+k_{4}M_{1}\frac{c_{1}}{c_{1}+IL_{10}}-d_{IL_{1}}IL_{1}, \end{array}$$

(4) $$\begin{array}{ccc} \frac{dIL_{10}}{dt} & = & k_{21}T\frac{c_{3}}{c_{3}+IL_{10}}+k_{5}M_{2}\frac{c_{2}}{c_{2}+IL_{10}}-d_{IL_{10}}IL_{10}, \end{array}$$

(5) $$\begin{array}{ccc} \frac{dIL_{17}}{dt} & = & k_{22}T\frac{c_{3}}{c_{3}+IL_{10}}-d_{IL_{17}}IL_{17}, \end{array}$$

(6) $$\begin{array}{ccc} \frac{dN}{dt} & = & k_{18}M_{d}+k_{19}\frac{IL_{17}}{IL_{17}+c_{IL_{7}}}-\mu_{N}N, \end{array}$$

(7) $$\begin{array}{ccc} \frac{dM_{0}}{dt} & = & k_{6}M_{d}-k_{7}M_{0}\frac{IL_{1}}{IL_{1}+c_{IL_{1}M_{0}}}-k_{8}M_{0}\frac{IL_{10}}{IL_{10}+c_{IL_{10}M_{0}}}\\ & & -k_{20}M_{0}\frac{IL_{17}}{IL_{17}+c_{IL_{17}M_{0}}}-\mu_{M}M_{0}, \end{array}$$

(8) $$\begin{array}{ccc} \frac{dM_{1}}{dt} & = & k_{7}M_{0}\frac{IL_{1}}{IL_{1}+c_{IL_{1}M_{0}}}+k_{20}M_{0}\frac{IL_{17}}{IL_{17}+c_{IL_{17}M_{0}}}-k_{9}M_{1}-\mu_{M}M_{1}, \end{array}$$

(9) $$\begin{array}{ccc} \frac{dM_{2}}{dt} & = & k_{8}M_{0}\frac{IL_{10}}{IL_{10}+c_{IL_{10}M_{0}}}+k_{9}M_{1}-\mu_{M}M_{2}, \end{array}$$

(10) $$\begin{array}{ccc} \frac{dC}{dt} & = & k_{10}F\frac{IL_{10}}{IL_{10}+c_{IL_{10}C}}-k_{11}C\frac{IL_{1}}{IL_{1}+c_{IL_{1}C}}, \end{array}$$

(11) $$\begin{array}{ccc} \frac{dF}{dt} & = & k_{12}F\frac{IL_{10}}{IL_{10}+c_{IL_{10}F}}, \end{array}$$

(12) $$\begin{array}{ccc} \frac{dS}{dt} & = & -\mu_{S}S, \end{array}$$

(13) $$\begin{array}{ccc} \frac{dT}{dt} & = & k_{13}M_{d}-\mu_{T}T. \end{array}$$

In the above “non-treatment” model, the equations model the dynamics of various entities, including the primary variables, which comprise cardiomyocytes $\left(M_{c}\right)$, dead cardiomyocytes $\left(M_{d}\right)$, interleukin 1 $\left(IL_{1}\right)$ cytokines, interleukin 10 $\left(IL_{10}\right)$ cytokines, interleukin 17 $\left(IL_{17}\right)$ cytokines, neutrophils (N), monocytes $\left(M_{0}\right)$, classically activated macrophages $\left(M_{1}\right)$, alternatively activated macrophages $\left(M_{2}\right)$, collagen (C), fibroblasts (F), stem cells (S), and γαT cells (T).

The principles of mass action kinetics, feedback regulation, and decay and mortality form the cornerstone of understanding complex biological and chemical systems. Mass action kinetics demonstrates that the rate of a chemical reaction is directly proportional to the concentration of reactants, providing a foundation for predicting reaction behavior [24]. Feedback regulation, by contrast, introduces dynamic complexity through nonlinear terms, as certain cytokines either inhibit or promote the production of others, generating intricate feedback loops that can amplify or dampen responses [25]. Lastly, decay and mortality account for natural degradation or death within the system, which is crucial for modeling long-term behavior [26]. Equation (1) describes the rate of change with respect to the time of $M_{c}$. The term $-k_{15}M_{c}\frac{IL_{17}}{IL_{17}+c_{IL_{17}M_{c}}}$ represents the effect of $IL_{17}$ on the destruction of $M_{c}$. It is derived from mass action laws with saturation kinetics modeled by the Michaelis–Menten form ($\frac{IL_{17}}{IL_{17}+c_{IL_{17}M_{c}}}$). The term $-k_{16}NM_{c}$ accounts for depletion of $M_{c}$ due to interactions with *N*. The term $-\mu_{M_{c}}M_{c}$ represents the natural mortality or degradation of $M_{c}$. Equation (2) describes the rate of change with respect to the time of the density of dead cardiomyocytes $M_{d}$. The term $k_{15}M_{c}\frac{IL_{17}}{IL_{17}+c_{IL_{17}M_{c}}}$ matches the depletion term for $M_{c}$, representing differentiation of $M_{c}$ into $M_{d}$. The term $\mu_{M_{c}}M_{c}$ reflects $M_{c}$ differentiation from degraded $M_{c}$. The last terms $-k_{2}M_{1}M_{d}-k_{17}NM_{d}$ capture interactions between $M_{d}$, $M_{1}$, and *N*, following mass action laws. Equation (3) describes the rate of change with respect to the time of $IL_{1}$. The term $k_{3}M_{d}$ represents secretion by $M_{d}$, and $k_{4}M_{1}\frac{c_{1}}{c_{1}+IL_{10}}$ represents secretion with the inhibition of the secretion rate by $IL_{10}$. The decay term $-d_{IL_{1}}IL_{1}$ represents natural degradation. Equations (4) and (5) describe the rate of change with respect to the time of $IL_{10}$ and $IL_{17}$, respectively. Similar terms describe $IL_{10}$ and $IL_{17}$ but with sources including *T*, $M_{2}$ for $IL_{10}$, and *T* for $IL_{17}$, respectively. Equation (6) describes the rate of change with respect to the time of the density of *N*. The term $k_{18}M_{d}$ represents migration of $M_{d}$ and the term $k_{19}\frac{IL_{17}}{IL_{17}+c_{IL_{7}}}$ represents recruitment of *N* by $IL_{17}$ following saturable kinetics. The term $-\mu_{N}N$ represents apoptosis of *N*. Equation (7) describes the rate of change with respect to the time of the density of $M_{0}$. The term $k_{6}M_{d}$ represents migration of $M_{d}$. Terms $-k_{7}M_{0}\frac{IL_{1}}{IL_{1}+c_{IL_{1}M_{0}}}$, $-k_{8}M_{0}\frac{IL_{10}}{IL_{10}+c_{IL_{10}M_{0}}}$, $-k_{20}M_{0}\frac{IL_{17}}{IL_{17}+c_{IL_{17}M_{0}}}$ show how cytokines $IL_{1},\;IL_{10},\;IL_{17}$ influence $M_{0}$ activation, with the saturation effects being described by constants. Equation (8) describes the rate of change with respect to the time of the density of $M_{1}$. The positive terms reflect the influences of cytokines $IL_{1}$ and $IL_{17}$ on $M_{1}$ by $M_{0}$, and the negative terms describe the natural decay of $M_{1}$. Equation (9) describes the rate of change with respect to the time of the density of $M_{2}$. The positive term $k_{9}M_{1}$ reflects the transformation from $M_{1}$ to $M_{2}$, while the negative term $\mu_{M}M_{2}$ accounts for the emigration $M_{2}$. Equations (10) and (11) describe the rate of change with respect to the time of the density of collagen (C) and the rate of change with respect to the time of the density of *F*, respectively. The first equation describes how *C* is influenced by *F* and $IL_{10}$, and it is degraded due to the presence of $IL_{1}$, while the second describes how *F* is produced in response to $IL_{10}$. Equation (12) describes the rate of change with respect to the time of *S*, which dies in the infarct region just like all other cells when their oxygen supply is cut off. Equation (13) describes the rate of change with respect to the time of *T*, which increases by production and decreases by death.

**Figure 1:**
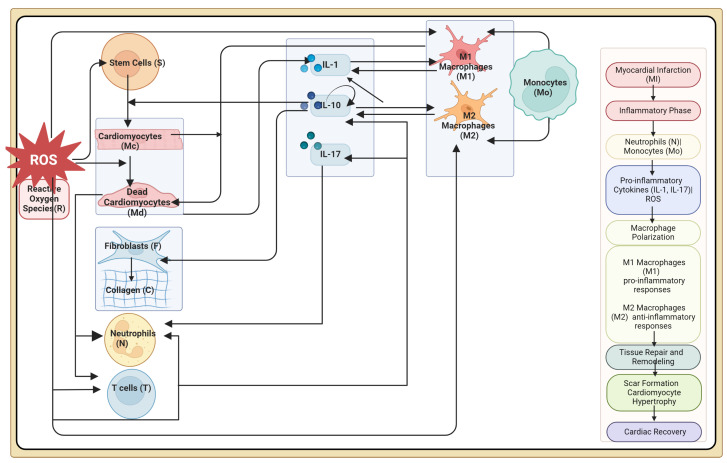
Schematic illustration of the intricate cellular and molecular dynamics involved in the post myocardial infarction (MI) cardiac recovery process.

**Figure 2:**
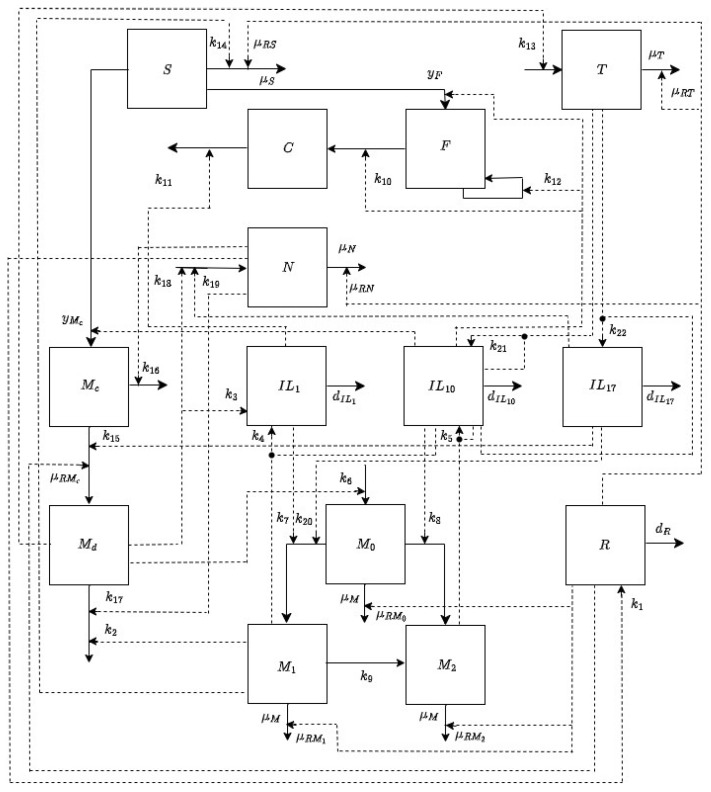
Flow diagram of the cellular and molecular interactions during the post-MI cardiac recovery process. Dashed lines represent interactions between two different populations of cells, while solid lines represent actual transfers of cells from one group to another. The primary variables considered in the diagram are cardiomyocytes: $M_{c}$, dead cardiomyocytes: $M_{d}$, interleukin 1: $IL_{1}$, interleukin 10: $IL_{10}$, interleukin 17: $IL_{17}$, neutrophils: *N*, monocytes: $M_{0}$, classically activated macrophages: $M_{1}$, alternatively activated macrophages: $M_{2}$, stem cells: *S*, γδT cells: *T*, and reactive oxygen species: *R*.

Figure 1 illustrates the cellular and molecular dynamics during the post-MI cardiac recovery process, while Figure 2 is a detailed flow diagram of all the corresponding interactions. The primary variables are cardiomyocytes $\left(M_{c}\right)$, dead cardiomyocytes $\left(M_{d}\right)$, interleukin 1 $\left(IL_{1}\right)$ cytokines, interleukin 10 $\left(IL_{10}\right)$ cytokines, interleukin 17 $\left(IL_{17}\right)$ cytokines, neutrophils (N), monocytes $\left(M_{0}\right)$, classically activated macrophages $\left(M_{1}\right)$, alternatively activated macrophages $\left(M_{2}\right)$, collagen (C), fibroblasts (F), stem cells (S), γαT cells (T), and ROSs (R). In the diagram, solid lines indicate an actual cell transfer from one group to another, such as the phenotypic shift from classically to alternatively activated macrophages, while dashed lines depict an interaction between two distinct populations of cells, such as the release of cytokines by macrophages.

The dynamics of the system that occur after medical treatment, such as oxygen restoration and subsequent injection of stem cells at the injury site, are taken into account in the second and third stages of the remodeling process.

The corresponding “treatment” model, based on the flow diagram in Figure 2, is as follows:

(14) $$\begin{array}{ccc} \frac{dM_{c}}{dt} & = & y_{M_{c}}S\frac{IL_{10}}{IL_{10}+m_{M_{c}}}-k_{15}M_{c}\frac{IL_{17}}{IL_{17}+c_{IL_{17}M_{c}}}-k_{16}NM_{c}\\ & & -\gamma \mu_{RM_{c}}\frac{R}{R+c_{RM_{c}}}M_{c}, \end{array}$$

(15) $$\begin{array}{ccc} \frac{dM_{d}}{dt} & = & k_{15}M_{c}\frac{IL_{17}}{IL_{17}+c_{IL_{17}M_{c}}}+\gamma \mu_{RM_{c}}\frac{R}{R+c_{RM_{c}}}M_{c}\\ & & -k_{2}M_{1}M_{d}-k_{17}NM_{d}, \end{array}$$

(16) $$\begin{array}{ccc} \frac{dIL_{1}}{dt} & = & k_{3}M_{d}+k_{4}M_{1}\frac{c_{1}}{c_{1}+IL_{10}}-d_{IL_{1}}IL_{1}, \end{array}$$

(17) $$\begin{array}{ccc} \frac{dIL_{10}}{dt} & = & k_{21}T\frac{c_{3}}{c_{3}+IL_{10}}+k_{5}M_{2}\frac{c_{2}}{c_{2}+IL_{10}}-d_{IL_{10}}IL_{10}, \end{array}$$

(18) $$\begin{array}{ccc} \frac{dIL_{17}}{dt} & = & k_{22}T\frac{c_{3}}{c_{3}+IL_{10}}-d_{IL_{17}}IL_{17}, \end{array}$$

(19) $$\begin{array}{ccc} \frac{dN}{dt} & = & \delta k_{18}M_{d}+k_{19}\frac{IL_{17}}{IL_{17}+c_{IL_{17}}}-\mu_{N}N-\gamma \mu_{RN}\frac{R}{R+c_{RN}}N, \end{array}$$

(20) $$\begin{array}{ccc} \frac{dM_{0}}{dt} & = & k_{6}M_{d}-k_{7}M_{0}\frac{IL_{1}}{IL_{1}+c_{IL_{1}M_{0}}}-k_{8}M_{0}\frac{IL_{10}}{IL_{10}+c_{IL_{10}M_{0}}}\\ & & -k_{20}M_{0}\frac{IL_{17}}{IL_{17}+c_{IL_{17}M_{0}}}-\mu_{M}M_{0}-\gamma \mu_{RM_{0}}\frac{R}{R+c_{RM_{0}}}M_{0}, \end{array}$$

(21) $$\begin{array}{ccc} \frac{dM_{1}}{dt} & = & k_{7}M_{0}\frac{IL_{1}}{IL_{1}+c_{IL_{1}M_{0}}}+k_{20}M_{0}\frac{IL_{17}}{IL_{17}+c_{IL_{17}M_{0}}}-k_{9}M_{1}\\ & & -\mu_{M}M_{1}-\gamma \mu_{RM_{1}}\frac{R}{R+c_{RM_{1}}}M_{1}, \end{array}$$

(22) $$\begin{array}{ccc} \frac{dM_{2}}{dt} & = & k_{8}M_{0}\frac{IL_{10}}{IL_{10}+c_{IL_{10}M_{0}}}+k_{9}M_{1}-\mu_{M}M_{2}\\ & & -\gamma \mu_{RM_{2}}\frac{R}{R+c_{RM_{2}}}M_{2}, \end{array}$$

(23) $$\begin{array}{ccc} \frac{dC}{dt} & = & k_{10}F\frac{IL_{10}}{IL_{10}+c_{IL_{10}C}}-k_{11}C\frac{IL_{1}}{IL_{1}+c_{IL_{1}C}}, \end{array}$$

(24) $$\begin{array}{ccc} \frac{dF}{dt} & = & y_{F}S\frac{IL_{10}}{IL_{10}+m_{F}}+k_{12}F\frac{IL_{10}}{IL_{10}+c_{IL_{10}F}}, \end{array}$$

(25) $$\begin{array}{ccc} \frac{dS}{dt} & = & -y_{M_{c}}S\frac{IL_{10}}{IL_{10}+m_{M_{c}}}-y_{F}S\frac{IL_{10}}{IL_{10}+m_{F}}-k_{14}M_{1}S\\ & & -\mu_{S}S-\gamma \mu_{RS}\frac{R}{R+c_{RS}}S, \end{array}$$

(26) $$\begin{array}{ccc} \frac{dT}{dt} & = & k_{13}M_{d}-\mu_{T}T-\gamma \mu_{RT}\frac{R}{R+c_{RT}}T, \end{array}$$

(27) $$\begin{array}{ccc} \frac{dR}{dt} & = & \omega k_{1}N-d_{R}R. \end{array}$$

The new treatment model is given by (14)–(27). In Equation (14), the term $y_{M_{c}},S\frac{IL_{10}}{IL_{10}+m_{M_{c}}}$ describes the production or activation of $M_{c}$ influenced by *S* and $IL_{10}$. The fraction $\frac{IL_{10}}{IL_{10}+m_{M_{c}}}$ is a Michaelis–Menten form, which shows a saturating effect of $IL_{10}$ on $M_{c}$. As $IL_{10}$ increases, its effect on $M_{c}$ saturates. The term $-\gamma \mu_{RM_{c}}\frac{R}{R+c_{RM_{c}}}M_{c}$ represents a decay or suppression process for $M_{c}$ mediated by *R*. The fraction $\frac{R}{R+c_{RM_{c}}}$ is another Michaelis–Menten form, showing that as *R* increases, its suppressive effect on $M_{c}$ saturates. The rate of this suppression is governed by $\mu_{RM_{c}}$ and modulated by γ, a scaling factor. In Equation (15), the term $\gamma \mu_{RM_{c}}\frac{R}{R+c_{RM_{c}}}M_{c}$ represents an alternative pathway by which $M_{c}$ contributes to $M_{d}$, mediated by *R*. The fraction $\frac{IL_{10}}{IL_{10}+m_{M_{c}}}$ shows that *R* enhances this production with saturation as *R* increases. The parameters γ and $\mu_{RM_{c}}$ modulate the rate of this contribution. In Equation (17), $IL_{10}$ is secreted by *T*, where the parameter $k_{21}$ denotes the secretion rate. A decreasing function is used to represent self-inhibition by $IL_{10}$, where $c_{3}$ denotes the self-inhibition parameter. In Equations (19)–(22), the terms $-\gamma \mu_{RN}\frac{R}{R+c_{RN}}N$, $-\gamma \mu_{RM_{0}}\frac{R}{R+c_{RM_{0}}}M_{0}$, $-\gamma \mu_{RM_{1}}\frac{R}{R+c_{RM_{1}}}M_{1}$, and $-\gamma \mu_{RM_{2}}\frac{R}{R+c_{RM_{2}}}M_{2}$ model the suppression or decay of *N*, $M_{0}$, $M_{1}$, and $M_{2}$ influenced by the regulatory molecule *R*, respectively. In Equation (24), *F* increases during treatment as *S* is stimulated by $IL_{10}$ to differentiate into *F* and decreases due to ROSs. In Equation (25), *S* decreases by differentiation and because of ROSs. Parameter $k_{14}$ represents apoptotic rate of *S* due to $M_{1}$, respectively. In Equation (26), *T* decreases because of ROSs. Equation (27) describes the rate of change with respect to the time of *R*, which increases by neutrophil production and decreases due to decay.

Note that in Model (14)–(27), the parameter γ accounts for the affect of risk factors, such as smoking, drinking, stress, and an unhealthy diet, on the level of ROS damage exerted on cells. The parameter ω in Equation (27) accounts for the increase in ROS production by neutrophils due to the increase in the level of oxygen restoration, during treatment, that is above the minimum necessary oxygen cell-survival level. The third parameter δ in Equation (19) accounts for the difference in the levels of neutrophil recruitment to the MI injury site due to an overactive or underactive immune system, such as that of individuals with autoimmune diseases or undergoing cancer treatments.

## 3. Analysis and Results

### 3.1. Sensitivity Analysis

All the rates and other parameters involved in the model have variations due to differences in each human or animal, differences in the cells of each individual and in their environment, and due to different measuring techniques and experimental errors. Therefore, it is important to determine how changes in each parameter affect the behavior of the model. Sensitivity analysis (SA) provides a quantitative approach to investigate the effects of parameter uncertainty on model outputs. Global sensitivity analysis (GSA) techniques investigate the effects of concurrent parameter changes on large but finite ranges and allow the investigation of interactions between parameters [27].

**Figure 3:**
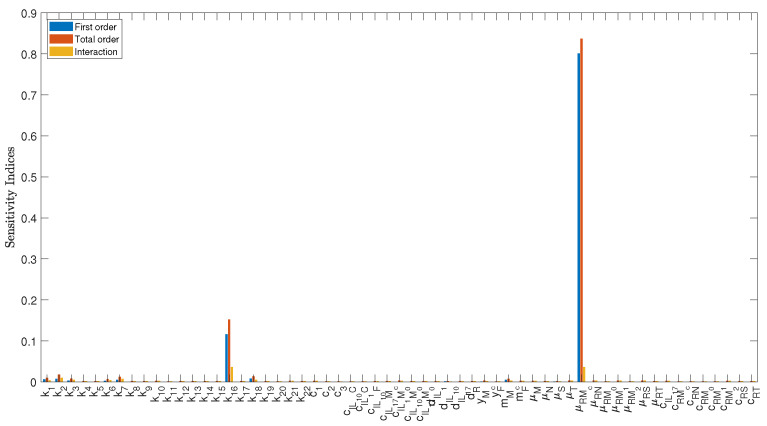
The eFAST sensitivity analysis for Model (14)–(27), displaying the indices for cardiomyocytes ($M_{c}$) with respect to all model parameters. The first-order sensitivity index measures how much one particular parameter contributes to the changes in $M_{c}$ keeping all other parameters constant, while the total order sensitivity index measures how $M_{c}$ changes when all model parameters are varied; therefore, incorporating the interactions between them. The interaction index for each input factor is obtained by subtracting the first order sensitivity index from the total order sensitivity index. The sensitivity analysis reveals the importance of the ROS destruction rate of $M_{c}$ ($\mu_{RM_{c}}$) and the destruction rate of $M_{c}$ by *N* ($k_{16}$).

We use the Extended Fourier Amplitude Sensitivity Test (eFAST), which is one of GSA methods and is proven to be one of the most reliable methods of variance-based techniques. In this method, the input parameters are varied to produce changes in the model outputs; the method calculates the contribution of each parameter in the determination of model output [28]. GSA is performed to identify the most important parameters of Model (14)–(27) and to characterize their influence on the numerical simulations. There is one sensitivity index for each output with respect to each parameter. To reduce their number by a factor of 14, we chose to calculate only the indices of cardiomyocytes since their number is the most important output. Moreover, the indices are functions of time, so we are only reporting their average over a simulation period of 30 days. Assuming uniform distributions, all parameters fluctuate according to the precise ranges and baseline values specified in Table 1, with $M_{c}\left(0\right)=4\times 10^{7}$, $M_{d}\left(0\right)=0$, $IL_{1}\left(0\right)=0.1$, $IL_{10}\left(0\right)=0.01$, $IL_{17}\left(0\right)=0.1$, $N_{0}\left(0\right)=0$, $M_{0}\left(0\right)=2\times 10^{3}$, $M_{1}\left(0\right)=0$, $M_{2}\left(0\right)=0$, $C\left(0\right)=839.5\times 10^{9}$, $F\left(0\right)=1\times 10^{8}$, $S\left(0\right)=2\times 10^{7}$, T(0)=0, and R(0)=210 are the initial conditions. The eFAST sensitivity analysis results are shown in Figure 3. From Figure 3, the parameters with highest total order effects are $\mu_{RM_{c}}$ and $k_{16}$. This implies that the cardiomyocytes ($M_{c}$) recovery after MI is heavily impacted by changes and uncertainty in the parameters that characterize the effectiveness of the ROS destruction rate of $M_{c}$ ($\mu_{RM_{c}}$), and the destruction rate of $M_{c}$ by *N* ($k_{16}$).

### 3.2. Numerical Simulations

To illustrate the functionality of the new model that was proposed, we perform a series of numerical simulations for the case of mild MI and severe MI. The severe MI is characterized by a significantly greater constant death rate of cardiomyocytes as well as an increased activation rate of cardiomyocytes due to $IL_{10}$. Because severe MI causes more tissue damage than moderate MI, there is a need for faster tissue regeneration, as seen by the higher activation rate. The values of the model parameters employed vary depending on whether the system represents humans, rats, mice, or another species. The parameters used in the numerical simulations, with their descriptions, experimental values, units, and references, are listed in Table 1. The initial conditions of the cardiomyocytes, cytokines, neutrophils, monocytes, macrophages, collagen densities, fibroblasts, stem cells, γαT cells and ROS densities are chosen as $M_{c}\left(0\right)=4\times 10^{7}$ cells/mL [31], $M_{d}\left(0\right)=0$ cells/mL (estimated), $IL_{1}\left(0\right)=0.1$ pg/mL [7], $IL_{10}\left(0\right)=0.01$ pg/mL (estimated), $IL_{17}\left(0\right)=0.1$ pg/mL (estimated), $N_{0}\left(0\right)=0$ cells/mL (estimated) $M_{0}\left(0\right)=2\times 10^{3}$ cells/mL, $M_{1}\left(0\right)=0$ cells/mL, $M_{2}\left(0\right)=0$ cells/mL [7], $C\left(0\right)=839.5\times 10^{9}$ pg/mL [6], $F\left(0\right)=1\times 10^{8}$ cells/mL (estimated), $S\left(0\right)=2\times 10^{7}$ cells/mL [32], T(0)=0 cells/mL (estimated), R(0)=210 pg/mL (estimated). All simulations are obtained using the adaptive MATLAB solver ode23s.

**Figure 4:**
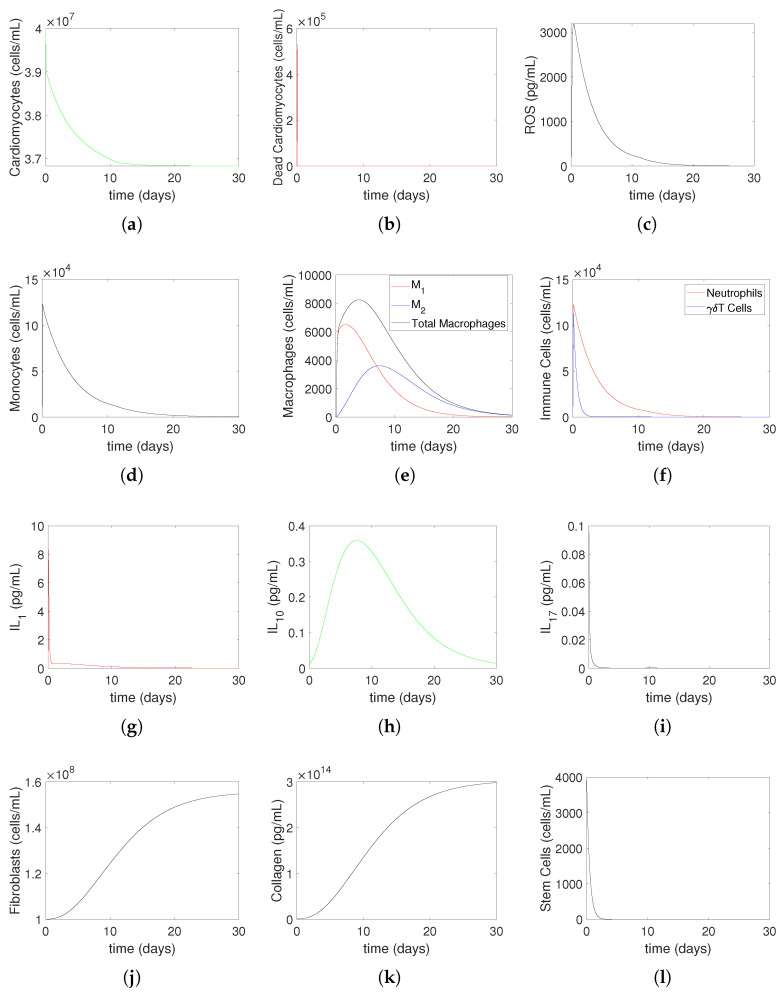
Mild MI without medical treatment: The evolution of heart muscle tissue and immune system cells to 30 days for Model (14)–(27) with $\mu_{M_{c}}=0.3,\;y_{M_{c}}=0.9$. This process is non-treatment until hour 2, followed by oxygen restoration at 2 h post-MI. In (**a**), cardiomyocytes $\left(M_{c}\right)$ undergo a monotonic decrease as a result of apoptosis and cellular necrosis. Conversely, dead cardiomyocytes $\left(M_{d}\right)$, as shown in (**b**), first significantly increase before decreasing to zero. ROS (R) shown in (**c**) increase until 2 h, then decrease and go to zero. Monocytes ($M_{0}$), as depicted in (**d**), increase as a result of the initial increase in dead cardiomyocytes and subsequently decrease due to their differentiation into classically activated macrophages and alternatively activated macrophages. As seen in (**e**), classically activated ($M_{1}$) and alternatively activated ($M_{2}$) macrophages increase initially as a result of differentiating from monocytes. $M_{2}$ eventually overtakes $M_{1}$ due to phenotypic switching and then both $M_{1}$ and $M_{2}$ decrease as they leave the site. Shown in (**f**) are the immune cells (the neutrophil (*N*) and γδT cells) that increase until 2 h, and then decrease to zero. The cytokines, $IL_{1}$ (red line), $IL_{10}$ (green line), and $IL_{17}$ (black line) are shown in (**g**), (**h**) and (**i**), respectively. Fibroblasts (F) and collagen (C), shown in (**j**,**k**), respectively, increase during the simulation period, while stem cells (S), shown in (**l**), decrease quickly and go to zero. The initial conditions used in the simulations are $M_{c}\left(0\right)=4\times 10^{7},\;$ $M_{d}\left(0\right)=0,\;$ $IL_{1}\left(0\right)=0.1,\;$ $IL_{10}\left(0\right)=0.01,\;$ $IL_{17}\left(0\right)=0.1,\;$ N(0)=0, $M_{0}\left(0\right)=2\times 10^{3},\;$ $M_{1}\left(0\right)=0,\;$ $M_{2}\left(0\right)=0,\;$ $F\left(0\right)=1\times 10^{8},\;$ $C\left(0\right)=839.5\times 10^{9},\;$ $S\left(0\right)=2\times 10^{7},\;$ T(0)=0, R(0)=210. All parameter values are given in Table 1.

**Figure 5:**
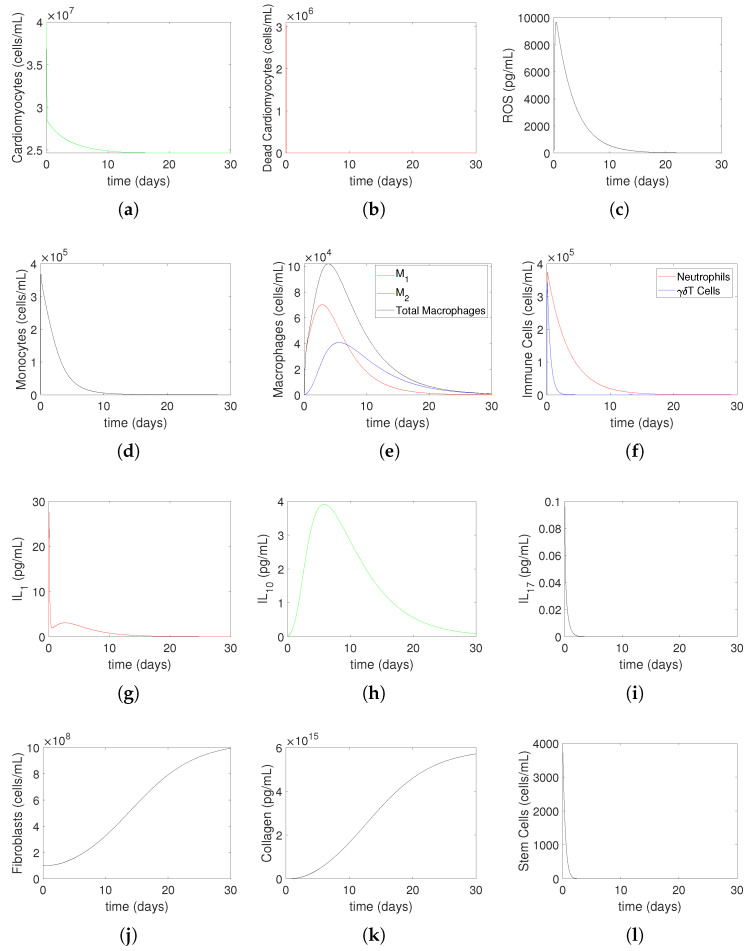
Severe MI without medical treatment: The evolution of heart muscle tissue and immune system cells to 30 days for Model (14)–(27) with $\mu_{M_{c}}=4.0677,\;y_{M_{c}}=2.52$. This process is non-treatment until hour 2, followed by oxygen restoration at 2 h post-MI. In (**a**), cardiomyocytes ($M_{c}$) steadily decline due to apoptosis and cellular necrosis. In contrast, the density of dead cardiomyocytes ($M_{d}$), shown in (**b**), initially rises sharply before gradually decreasing to zero. Reactive oxygen species (ROSs) (*R*), depicted in Figure (**c**), increase until hour 2, after which they decline and eventually reach zero. As shown in Figure (**d**), monocyte ($M_{0}$) levels rise in response to the initial surge in dead cardiomyocytes and later decrease as they differentiate into classically activated macrophages and alternatively activated macrophages. Figure (**e**) illustrates the dynamics of classically activated ($M_{1}$) and alternatively activated ($M_{2}$) macrophages, both of which increase initially due to monocyte differentiation. Over time, $M_{2}$ surpasses $M_{1}$ due to phenotypic switching, and eventually, both macrophage types decline as they exit the site. Immune cells, including neutrophils (*N*) and γδT cells, are shown in Figure (**f**). Their numbers increase until hour 2, after which they decline to zero. The cytokines $IL_{1}$ (red line), $IL_{10}$ (green line), and $IL_{17}$ (black line) are represented in Figures (**g**), (**h**), and (**i**), respectively. Fibroblast (F) and collagen (C) levels, depicted in Figures (**j**) and (**k**), respectively, continue to rise throughout the simulation period. Meanwhile, stem cells (S), illustrated in Figure (**l**), rapidly decrease and eventually reach zero. The initial conditions used in the simulations are $M_{c}\left(0\right)=4\times 10^{7},\;$ $M_{d}\left(0\right)=0,\;$ $IL_{1}\left(0\right)=0.1,\;$ $IL_{10}\left(0\right)=0.01,\;$ $IL_{17}\left(0\right)=0.1,\;$ N(0)=0, $M_{0}\left(0\right)=2\times 10^{3},\;$ $M_{1}\left(0\right)=0,\;$ $M_{2}\left(0\right)=0,\;$ $F\left(0\right)=1\times 10^{8},\;$ $C\left(0\right)=839.5\times 10^{9},\;$ $S\left(0\right)=2\times 10^{7},\;$ T(0)=0, R(0)=210. All parameter values are given in Table 1.

Initially, we numerically investigate the evolution of heart muscle tissue and immune system cells for 30 days after MI for Model (14)–(27) with $\mu_{M_{c}}=0.3,\;y_{M_{c}}=0.9$ and $\mu_{M_{c}}=4.0677,\;y_{M_{c}}=2.52$ for mild MI (Figure 4) and for severe MI (Figure 5), respectively. This process represents non-treatment until hour 2, followed by oxygen restoration at 2 h post-MI. Figure 4 and Figure 5 present simulated solutions that predict the temporal evolution of cardiac tissue components, immune cell populations, and signaling molecules during the 30-day post-MI healing process for mild MI and severe MI, respectively. Each subfigure corresponds to a critical aspect of the injury response, providing an insight into both early inflammatory dynamics and longer-term reparative processes. Below, the meaning of each subfigure is discussed in detail, along with comparisons to experimental and clinical data where available. In part (a), the model predicts a monotonic decrease in the cardiomyocyte population due to apoptosis and necrosis. This decline reflects the primary impact of ischemia on cardiac muscle cells and is a hallmark of MI. Real data from histological studies of infarcted hearts show a similar pattern, with about 25% of cardiomyocytes lost within a few hours post-MI [33]. The long-term depletion of cardiomyocytes contributes to impaired cardiac function, consistent with clinical observations. In part (b), dead cardiomyocytes initially increase sharply due to widespread cell death triggered by ischemia. Over time, this population decreases to zero as necrotic debris is cleared by macrophages. Experimental studies confirm this trajectory, showing a peak in necrotic tissue during the first few days after MI, followed by gradual clearance mediated by immune cells [19]. In part (c), ROS levels rise steeply during the first 2 h due to ischemia-induced oxidative stress. After oxygen restoration, ROS decline and eventually return to baseline. In part (d), monocyte recruitment increases in response to signals from dead cardiomyocytes. Their numbers peak as monocytes infiltrate the injured myocardium and then decrease as they differentiate into macrophages. This prediction corresponds to in vivo findings that monocyte levels peak around 3 days [21] post-MI and decline as they transition to reparative roles. In part (e), the differentiation of monocytes into classically activated M1 and alternatively activated M2 is captured in this plot. Initially, M1 macrophages dominate, driving inflammation, but M2 macrophages surpass M1 over time due to phenotypic switching. This transition is a critical part of the healing process, as M2 macrophages promote tissue repair and fibrosis. M1 macrophages are able to switch to M2 macrophages at a constant rate [7]. In part (f), immune cells (the neutrophil and γδT cells) increase rapidly within the first 2 h post-MI, initiating the inflammatory response, and then decrease as inflammation resolves. After an MI, the number of neutrophils that have moved to the infarct area peaks between 1 and 3 days later and begins to rapidly decrease on day 5 [17], showing that neutrophils peak early in the acute phase and are cleared within days. In part (g), IL–1 rises sharply, reflecting its role as a pro-inflammatory cytokine released in response to necrotic cell debris. This increase aligns with the clinical data, showing elevated IL–1 levels during the acute inflammatory phase of an MI. Supporting experimental data from in mice models indicate that IL–1 levels increase within the first 3 h, peak at 6 h, and decrease by 24 h post-MI [34]. These findings highlight IL–1’s pivotal role in initiating inflammation and its potential as a therapeutic target in modulating early immune responses following an MI. In part (h), IL–10 increases later, promoting the resolution of inflammation and repair. In part (i), IL–17 exhibits a distinct trajectory, reflecting its dual role in promoting inflammation and influencing tissue repair. Its behavior aligns with studies that report increased IL–17 expression in patients with an MI, especially during the transition from inflammation to healing. In part (j), the fibroblast (*F*) population increases steadily throughout the simulation, reflecting their role in extracellular matrix (ECM) deposition and scar formation [17]. This trend corresponds to experimental observations showing fibroblast proliferation and migration into the infarct zone within days of an MI, persisting throughout the healing phase. In part (k), collagen levels (*C*) rise in tandem with fibroblast activity, representing the formation of a fibrotic scar to replace lost myocardium. In part (l), stem cells (*S*) decrease rapidly.

**Figure 6:**
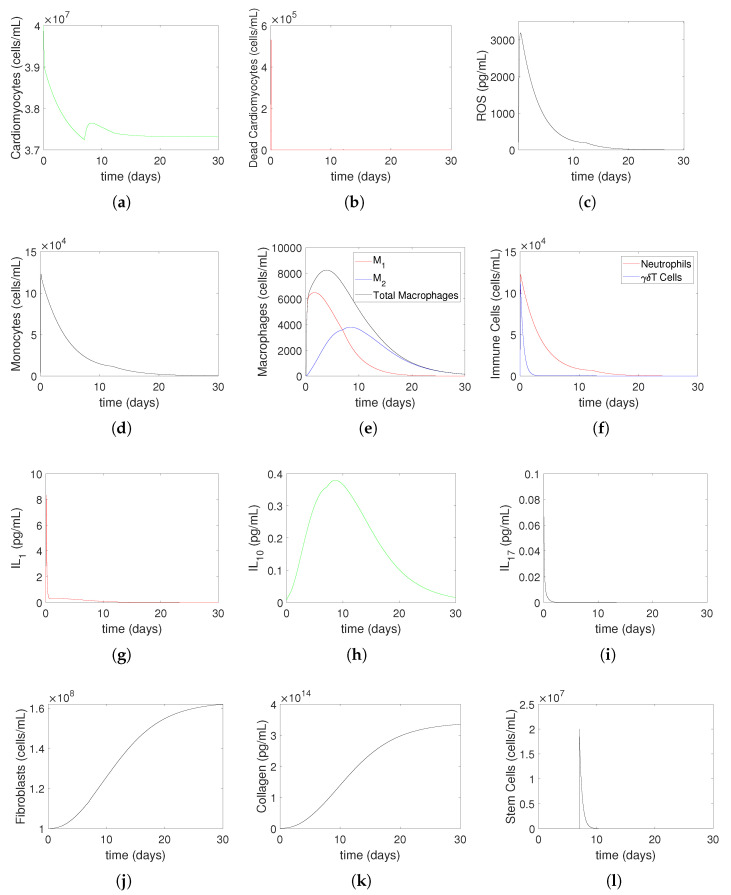
Mild MI with medical treatment: The evolution of heart muscle tissue and immune system cells to 30 days for Model (14)–(27). This process is non-treatment until hour 2, oxygen restoration at 2 h post-MI, followed by a stem cell injection at 7 days post-MI. Cardiomyocytes ($M_{c}$) shown in (**a**) first decrease until hour 2, then increase slightly from hour 2 to day 7, followed by an increase until stabilizing at a constant post-MI recovery level. Dead cardiomyocytes ($M_{d}$) density shown in (**b**) is less than that in the numerical simulation with the non-treatment model [9], because the death of the cardiomyocytes is prevented with the treatment. Reactive oxygen species (ROSs) (*R*) shown in (**c**) increases until 2 h, at which point it goes to zero. The density value of monocytes ($M_{0}$) and the values and overall percentage composition of macrophages ($M_{1}$, $M_{2}$), shown in (**d**,**e**), are also different from the corresponding values in the non-treatment model simulations. Neutrophils (*N*) and γδT cells, which are immune cells shown in (**f**), increase until 2 h and then decrease to zero. The cytokines, $IL_{1}$ (red line), $IL_{10}$ (green line) and $IL_{17}$ (black line), are shown in (**g**), (**h**) and (**i**), respectively. Fibroblasts (F) and collagen (*C*), shown in (**j**) and (**k**), respectively, increase during the simulation period, while stem cells (S), shown in (**l**), decrease quickly and go to zero. The initial conditions used in the simulations are $M_{c}\left(0\right)=4\times 10^{7},\;$ $M_{d}\left(0\right)=0,\;$ $IL_{1}\left(0\right)=0.1,\;$ $IL_{10}\left(0\right)=0.01,\;$ $IL_{17}\left(0\right)=0.1,\;$ N(0)=0, $M_{0}\left(0\right)=2\times 10^{3},\;$ $M_{1}\left(0\right)=0,\;$ $M_{2}\left(0\right)=0,\;$ $F\left(0\right)=1\times 10^{8},\;$ $C\left(0\right)=839.5\times 10^{9},\;$ $S\left(0\right)=2\times 10^{7},\;$ T(0)=0, R(0)=210. All parameter values are given in Table 1.

**Figure 7:**
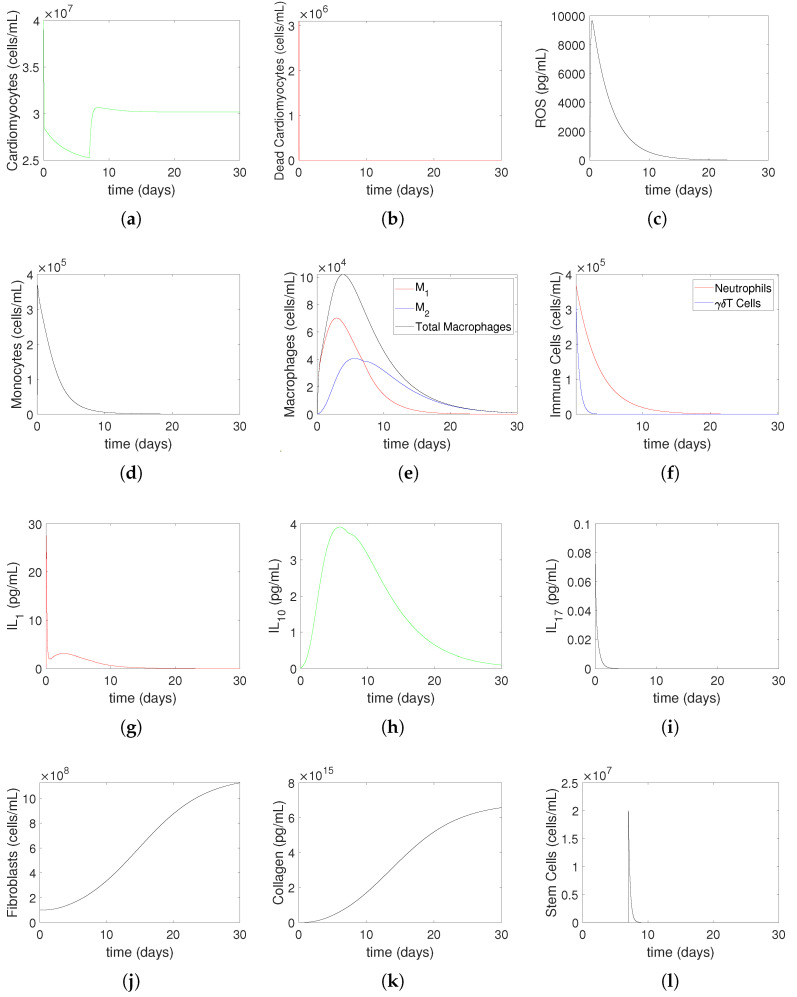
Severe MI with medical treatment: The evolution of heart muscle tissue and immune system cells to 30 days for Model (14)–(27). This process is non-treatment until hour 2, oxygen restoration at 2 h post-MI, followed by a stem cell injection at 7 days post-MI. Cardiomyocytes ($M_{c}$) in (**a**) initially decline until hour 2, then experience a slight increase from hour 2 to day 7, followed by a continuous rise until it stabilizes at a steady post-MI recovery level. The density of dead cardiomyocytes ($M_{d}$) in (**b**) is lower than that observed in the numerical simulation of the non-treatment model [9], as the treatment helps prevent cardiomyocyte death. Reactive oxygen species (ROSs) (*R*), depicted in (**c**), increase until hour 2, after which they decline to zero. The density of monocytes ($M_{0}$), along with the values and overall percentage composition of macrophages ($M_{1}$, $M_{2}$), shown in (**d**) and (**e**), respectively, also differ from those in the non-treatment model simulations. Neutrophils (*N*) and γδT cells, illustrated in (**f**), initially increase until hour 2 before decreasing to zero. The cytokines $IL_{1}$ (red line), $IL_{10}$ (green line), and $IL_{17}$ (black line) are represented in (**g**), (**h**), and (**i**), respectively. Fibroblasts (F) and collagen (C), shown in (**j**) and (**k**), respectively, continue to increase throughout the simulation, whereas stem cells (S), illustrated in Figure (**l**), rapidly decline and eventually reach zero. The initial conditions used in the simulations are $M_{c}\left(0\right)=4\times 10^{7},\;$ $M_{d}\left(0\right)=0,\;$ $IL_{1}\left(0\right)=0.1,\;$ $IL_{10}\left(0\right)=0.01,\;$ $IL_{17}\left(0\right)=0.1,\;$ N(0)=0, $M_{0}\left(0\right)=2\times 10^{3},\;$ $M_{1}\left(0\right)=0,\;$ $M_{2}\left(0\right)=0,\;$ $F\left(0\right)=1\times 10^{8},\;$ $C\left(0\right)=839.5\times 10^{9},\;$ $S\left(0\right)=2\times 10^{7},\;$ T(0)=0, R(0)=210. All parameter values are given in Table 1.

Figure 6 and Figure 7 illustrate the numerical simulation results of heart muscle tissue and immune system cell evolution over 30 days post-myocardial infarction (MI), using Model (14)–(27) for a mild MI (Figure 6) and a severe MI (Figure 7). The simulations incorporate a treatment strategy involving oxygen restoration at 2 h post-MI and stem cell injection at 7 days post-MI, shown experimentally [35] and numerically [10] to be optimal. These figures elucidate how treatment modifies tissue recovery and immune response dynamics compared to non-treatment scenarios. Under normal physiological conditions, small quantities of reactive oxygen species (ROSs) are produced during processes like aerobic respiration and inflammation [36]. These ROS levels remain below a baseline critical value $R_{c}$, causing no harm, as reflected in the model by setting the damage parameter γ=0 for ROS levels below $R_{c}$. However, during ischemic conditions, ROS levels exceed $R_{c}$, leading to tissue damage. This mechanism is central to the dynamics observed in the subplots. In part (a), the density of heart muscle cells decreases until hour 2 due to ischemia, stabilizes after oxygen restoration, and begins to recover slightly by day 7. Following stem cell injection, the density increases further, stabilizing at a higher post-MI recovery level. These trends are consistent with the experimental observations, showing that oxygen restoration halts ischemic damage and that stem cell therapy promotes myocardial regeneration. Part (b) shows that the density of dead cardiomyocyte peaks before treatment and remains lower than in non-treatment simulations [9], reflecting the protective effects of timely intervention. ROS dynamics in part (c) reveal a sharp increase until 2 h due to ischemic stress, followed by a rapid decline to zero after oxygen restoration, consistent with observed oxidative stress resolution upon reperfusion. Parts (d) and (e) illustrate immune cell dynamics, where monocyte density and macrophage composition reflect recruitment to the injury site and a shift toward reparative phenotypes. Similarly, part (f) shows neutrophils and γδT cells peaking during the acute inflammatory phase (by hour 2) and declining as inflammation resolves. Parts (g)–(i) depict cytokine dynamics, where $IL_{1}$ (pro-inflammatory) peaks early, $IL_{10}$ (anti-inflammatory) rises during recovery, and $IL_{17}$ increases steadily, reflecting scar formation. Fibroblasts and collagen dynamics, shown in parts (j) and (k), increase throughout the simulation period, indicating active tissue remodeling and scar stabilization, consistent with reparative processes observed post-MI. Finally, part (l) shows that stem cell density decreases rapidly after injection as they differentiate and integrate into the tissue.

Next, we numerically investigate the effects of the post-MI IR injury on the LV remodeling process for different values of the parameters δ, ω, and γ: δ=ω=γ=1 (baseline/normal levels), δ=ω=1,γ=10 (elevated risk factors), δ=10,ω=γ=1 (overactive immune system), and δ=1,ω=10,γ=1 (excessive oxygen restoration). Recall that in Model (14)–(27), the parameter γ accounts for the affect of risk factors, such as smoking, drinking, stress, and unhealthy diet, on the level of ROS damage exerted on cells; the parameter ω in Equation (27) accounts for the increase in ROS production by Neutrophils due to the increase in the level of oxygen restoration, during treatment, that is above the minimum necessary oxygen cell-survival level; and the parameter δ in Equation (19) accounts for the difference in levels of neutrophils’ recruitment to the MI injury site due to an overactive or underactive immune system, such as individuals with autoimmune diseases or undergoing cancer treatments.

**Figure 8:**
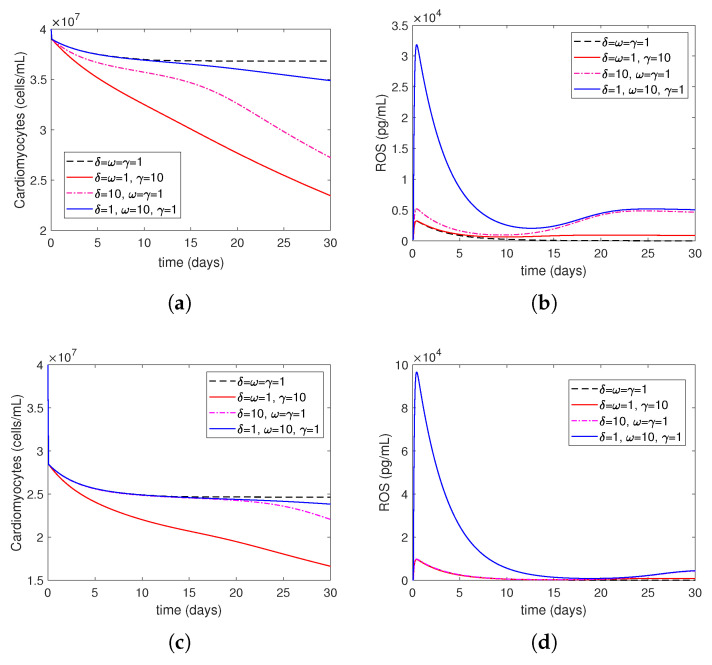
MI without treatment: cardiomyocytes ($M_{c}$) and reactive oxygen species (ROSs) during the first 30 days after MI with $\mu_{M_{c}}=0.3,\;y_{M_{c}}=0.9$ for mild MI, (**a**) cardiomyocytes, (**b**) ROSs and with $\mu_{M_{c}}=4.0677,\;y_{M_{c}}=2.52$ for severe MI, (**c**) cardiomyocytes, (**d**) ROSs, respectively.

Figure 8 shows the time evolution of cardiomyocytes and ROS levels after MI without medical treatment. In both mild and severe MI cases, there is a dramatic decrease during the first 2 h after oxygen restoration time followed by a subsequent decrease in cardiomyocytes. In both cases, there is a rapid increase in the level of ROSs in the first 2 h, and after oxygen restoration time, the decrease continues until about two weeks. After that, there is a large increase in mild MI, while the amount of increase in severe MI is small and becomes stable in both cases. As γ increases, cardiomyocytes decrease more while ω increases. In ROSs, the first two hours following the oxygen restoration time show a rise, which is followed by a sharp decline.

**Figure 9:**
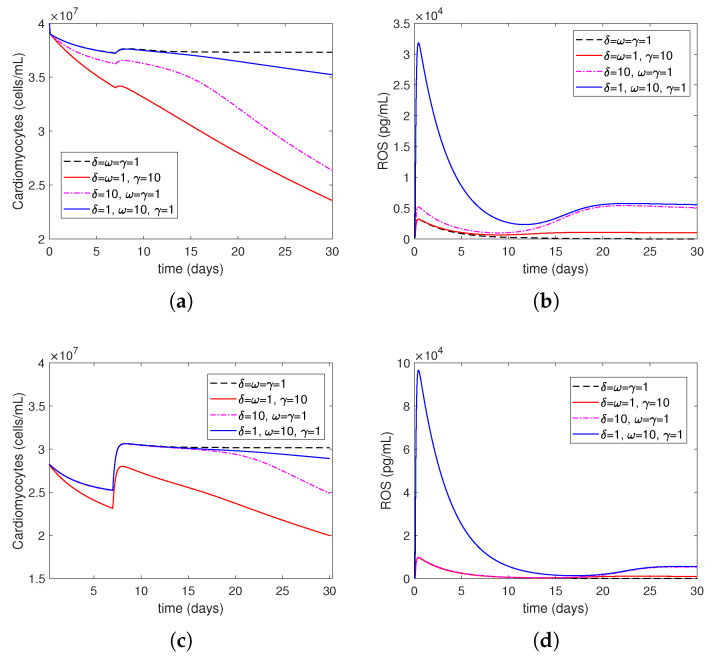
MI with stem-cell treatment at day 7: cardiomyocytes ($M_{c}$) and reactive oxygen species (ROSs) during the first 30 days after MI with $\mu_{M_{c}}=0.3,\;y_{M_{c}}=0.9$ for mild MI, (**a**) cardiomyocytes, (**b**) ROSs and with $\mu_{M_{c}}=4.0677,\;y_{M_{c}}=2.52$ for severe MI, (**c**) cardiomyocytes, (**d**) ROSs, respectively.

Figure 9 shows the time evolution of cardiomyocytes and ROS levels after an MI with medical treatment. This process is non-treatment until hour 2, oxygen restoration at 2 h post-MI, followed by a stem cell injection at 7 days post-MI.

Although there is a difference between the cardiomyocytes curves in mild MI in Figure 8a when no treatment is applied, during 20 days post MI, there is no difference in severe MI in Figure 8c, except the red curve, which has the largest decrease in the two cases, i.e., the worst one, containing elevated risk factors such as smoking, drinking, stress, and unhealthy diet. However, after 20 days, there is a slight difference in severe MI. When treatment is applied, in addition to the increase in cardiomyocytes levels in severe MI, the difference between the cardiomyocytes curves in Figure 9c after 20 days increases. As γ increases, cardiomyocytes decrease more. As ω increases, in ROSs, the first two hours following the oxygen restoration time show a rise, which is followed by a sharp decline.

## 4. Discussion

The presented study demonstrates that our mathematical model is able to capture post-MI events after mild and severe MI without stem cell treatment and with stem cell treatment. Without stem cell treatment, the model reported that acute MI results in a surge in dead cardiomyocytes, followed by a decrease, possibly due to macrophage-mediated clearance. ROS levels initially rise, indicating acute tissue damage, and then decline as inflammation resolves. Monocytes increase, differentiating into M1 and M2 macrophages, which drive inflammation and tissue remodeling. Neutrophils and γδT cells peak early, indicating their involvement in tissue damage-induced inflammation. Temporal patterns of interleukins (IL–1, IL–10, IL–17) correspond to inflammatory and tissue healing activities. Fibroblast and collagen levels rise, indicating tissue regeneration and scarring. Heart tissue-originated stem cells deplete due to inadequate oxygen and unfavorable extracellular matrix, limiting their repairing capability for the damaged tissue.

The model also shows that, following stem cell treatment, there is a noticeable increase in cardiomyocyte populations, indicating potential regeneration and repair of heart muscle. Stem cell therapy also reduces dead cardiomyocyte density, suggesting decreased heart muscle damage. ROS levels initially rise but then fall after stem cell treatment, indicating a potential release of antioxidant enzymes or paracrine chemicals by stem cells. Stem cell therapy also modulates cytokine production and release, influencing the inflammatory environment to support tissue repair and immunoregulation. Lastly, the model predicts much fewer fibroblasts and less collagen accumulation post-treatment, suggesting reduced scar formation and a microenvironment that promotes cardiac repair.

A major strength of this model is that it is sensitive to clinically relevant parameters, such as ω, γ, and δ. Recall that the parameter ω in Equation (26) accounts for the increase in ROS production by neutrophils due to the increase in the level of oxygen restoration during treatment, which is above the minimum necessary oxygen cell survival level; the parameter γ accounts for the effects of patient risk factors, such as smoking, drinking, stress, and unhealthy diet, on the level of ROS damage exerted on cells; the parameter δ in Equation (19) accounts for the difference in the levels of neutrophil recruitment to the MI injury site due to an overactive or underactive immune system, such as individuals with autoimmune diseases or undergoing cancer treatments. The numerical simulations (Figure 8 and Figure 9) show that our model is able to describe some important phenomena observed in clinical practices, which are summarized as follows:

- Increasing ω represents the surge in the level of oxygen restoration, which leads to worsened tissue damage due to elevated ROS levels. As explained by ω in Equation (27) in this article, ROS production by neutrophils increases due to an increase in circulating oxygen levels during treatment (i.e., above the minimum required level). This is consistent with reports in the scientific literature that an increased production of reactive oxygen species (ROSs) is one of the mechanisms responsible for mediating IR injury during reperfusion [2].
- Increasing γ represents increased ROS damage in response to heightened patient risk factors. Following Model (14)–(27), the parameter γ quantifies the influence of risk factors (such as smoking, drinking, stress, and bad food) on the levels of cellular ROS damage. According to the scientific literature, smoking, abdominal obesity, and hypertension are the primary variables that cause most cases of myocardial infarctions [37]. Another study that investigated mortality in the reperfusion era of acute myocardial infarction found that age had the greatest impact, and other significant factors, although less influential, included previous myocardial infarction, height, duration of treatment, diabetes, weight, smoking status, and stress [38].
- Increasing δ represents the higher level of neutrophil recruitment due to differences in the immune system. A higher δ value results in an overactive immune response, which consequently enhances neutrophil infiltration and tissue damage. This is shown in Equation (19), which considers variations in the extent of neutrophil recruitment to the location of myocardial infarction (MI) damage caused by an excessively active or insufficiently active immune system (as seen in persons with autoimmune disorders or receiving cancer therapy). The observed alterations in neutrophil levels correspond to the findings reported in the current literature. Neutrophils are recognized as one of the initial cells to arrive at infection locations [17,39]. When activated, these neutrophils secrete elastase and matrix metalloproteinases (MMPs), which help attract inflammatory cells to injured tissue and assist in removing dead cardiomyocytes. Simultaneously, deceased heart muscle cells stimulate the activation of γδT cells and the secretion of cytokines such IL–10 and IL–17 [11,40]. Specifically, IL–17A enhances the invasion of neutrophils and causes the death of cardiomyocytes during ischemia/reperfusion damage [41,42]. During the process of migration and phagocytosis, neutrophils produce reactive oxygen species (ROSs), which can worsen tissue damage and inflammation [43].

A limitation of the current model arises from the lack of clinical data needed to accurately determine several critical parameters. These parameters were estimated through assumptions and model fitting. The future availability of more comprehensive and detailed clinical data will enable the refinement of these parameters and allow the inclusion of additional facets of the complex biological processes that occur after an MI. For example, in order to guide treatment decisions and select patients who are most likely to benefit from blood flow restoration, the assessment of myocardial viability and scarring is still critical [44]. In the future, parameterization for varying levels of MI severity and other internal and external factors will help develop more accurate models tailored to individual patient profiles. Highly specific and accurate models capable of simulating realistic scenarios will empower researchers and clinicians to design better-targeted experiments. These models can reduce the reliance on costly and time-consuming cellular, animal and clinical studies, thus accelerating the development of innovative MI treatment strategies.

## 5. Conclusions

In this study, the task was to develop a comprehensive mathematical model that accounts for various post-myocardial infarction (MI) events and factors, including the inflammatory dynamics of γδT cells, IL–17 elements, neutrophils, and ROS-mediated ischemia-reperfusion (IR) injury, cardiac remodeling driven by necrosis and fibrosis, and stem cell therapy. This study presents a novel model that incorporates three clinically relevant parameters: the oxygen restoration rate (ω), patient risk factors (γ), and the neutrophil recruitment profile (δ). These additions enhance the model’s relevance and applicability to real-world clinical scenarios, making it a significant advancement over the previously published model [10]. The developed model effectively addresses several key challenges in understanding post-MI responses. It highlights the critical importance of the oxygen restoration rate, showing that while oxygen restoration is essential for reperfusing the affected heart tissue, an excessive rate can lead to reperfusion injury due to the generation of excessive reactive oxygen species (ROSs). This finding underscores the delicate balance required in medical interventions to optimize patient outcomes without causing additional damage. Moreover, the model demonstrates how increased patient risk factors, such as smoking, drinking, and obesity, exacerbate ROS damage and worsen reperfusion tissue damage. These insights align with clinical observations and provide a quantitative framework for understanding how lifestyle factors can influence MI recovery. By incorporating these risk factors, the model offers a more personalized approach to predicting patient outcomes and tailoring treatment strategies accordingly. Furthermore, the model underscores the significant role of immunodeficiency in post-MI recovery. It reveals that both an overactive and underactive immune response can lead to enhanced neutrophil infiltration and subsequent tissue damage. This finding is particularly important for understanding the variability in patient responses to an MI and developing targeted therapies to modulate the immune response appropriately. Overall, the mathematical model presented in this study offers valuable insights into MI pathology and provides a robust computational tool for researchers. It facilitates the development of treatment options and procedures aimed at reducing the adverse effects of IR injury on left ventricular (LV) remodeling, ultimately improving patient outcomes. By capturing the complex interplay of various factors and their influence on MI recovery, the model serves as a predictive tool that can guide clinical decision making and optimize therapeutic interventions. In conclusion, this study has successfully developed a sophisticated mathematical model that addresses the multifaceted nature of post-MI events. The inclusion of clinically relevant parameters enhances its applicability and relevance to real-world scenarios, making it a powerful tool for understanding MI pathology and guiding treatment strategies. The model’s ability to capture the sensitivity of patient responses to various factors, such as oxygen restoration rate, risk factors, and immune response, provides a comprehensive framework for improving patient care and outcomes after MI.

## Figures and Tables

**Table 1:** Parameter values for the mathematical model.

Parameter	Description	Range	Value	Units	Ref
$k_{1}$	Production rate of ROSs	[0.2,0.4]	0.3	pg/cells/day	ES *
$k_{2}$	Rate at which $M_{d}$ are engulfed by $M_{1}$	$\left[10^{-2},0.1\right]$	0.09	mL/cells/day	[10]
$k_{3}$	Secretion rate of $IL_{1}$ by $M_{d}$	$\left[10^{-4},9\;\times \;10^{-4}\right]$	0.0004	pg/cells/day	[10]
$k_{4}$	Secretion rate of $IL_{1}$ by $M_{1}$	$\left[10^{-4},9\;\times \;10^{-4}\right]$	0.0005	pg/cells/day	[7]
$k_{5}$	Secretion rate of $IL_{1}$ by $M_{2}$	$\left[10^{-4},9\;\times \;10^{-4}\right]$	0.0005	pg/cells/day	[7]
$k_{6}$	Recruitment rate of $M_{0}$ based on $M_{d}$	[1,10]	4	1/day	[10]
$k_{7}$	$IL_{1}$ activation rate for $M_{1}$	[0.1,0.9]	0.7	1/day	[10]
$k_{8}$	$IL_{10}$ activation rate for $M_{2}$	[0.1,9]	0.3	1/day	[7]
$k_{9}$	Activation rate of $M_{1}$ to $M_{2}$	$\left[10^{-2},0.2\right]$	0.075	1/day	[7]
$k_{10}$	Production rate of *C* by *F*	$\left[10^{5},3\;\times \;10^{6}\right]$	$26\;\times \;10^{5}$	pg/cells/day	[6]
$k_{11}$	Degradation rate of *C* by $IL_{1}$	$\left[10^{-4},9\;\times \;10^{-4}\right]$	0.0003	1/day	[6]
$k_{12}$	Fibroblast growth rate	[0.1,0.9]	0.25	1/day	[6]
$k_{13}$	Recruitment rate of *T* based on $M_{d}$	[0,4]	4	1/day	ES *
$k_{14}$	Apoptotic rate of *S* due to $M_{1}$	$\left[9\;\times \;10^{-6},10^{-5}\right]$	0.00001	mL/cells/day	[10]
$k_{15}$	Destruction rate of $M_{c}$ by $IL_{17}$	$\left[10^{-4},10^{-3}\right]$	0.001	1/day	ES *
$k_{16}$	Destruction rate of $M_{c}$ by *N*	$\left[10^{-8},10^{-7}\right]$	$10^{-7}$	mL/cells/day	ES *
$k_{17}$	Rate at which $M_{d}$ are engulfed by *N*	$\left[10^{-7},10^{-6}\right]$	$10^{-6}$	mL/cells/day	ES *
$k_{18}$	Recruitment rate of *N* based on $M_{d}$	[3,4]	4	1/day	ES *
$k_{19}$	Recruitment rate of *N* based on $IL_{17}$	[0.5,1]	1	cells/mL/day	ES *
$k_{20}$	Activation rate of $IL_{17}$ to activate $M_{1}$	[0.5,0.7]	0.7	1/day	ES *
$k_{21}$	Secretion rate of $IL_{10}$ by γδT	$\left[10^{-7},10^{-6}\right]$	$10^{-6}$	pg/cells/day	ES *
$k_{22}$	Secretion rate of $IL_{17}$ by γδT	$\left[10^{-7},10^{-6}\right]$	$10^{-6}$	pg/cells/day	ES *
$c_{1}$	Effectiveness of $IL_{10}$ inhibition on $IL_{1}$	[21,25]	25	pg/mL	[7]
$c_{2}$	Effectiveness of $IL_{10}$ inhibition on $IL_{10}$	[95,100]	100	pg/mL	[7]
$c_{3}$	Effectiveness of $IL_{10}$ inhibition on $IL_{17}$	[95,100]	100	pg/mL	ES *
$c_{IL_{10}C}$	Effectiveness of $IL_{10}$ inhibition on *F*	[3,5]	5	pg/mL	[10]
$c_{IL_{1}C}$	Effectiveness of $IL_{1}$ promotion on *C*	[8,10]	10	pg/mL	[10]
$c_{IL_{10}F}$	Effectiveness of $IL_{10}$ promotion on *F*	[2,2.5]	2.5	pg/mL	[10]
$c_{IL_{17}M_{c}}$	Effectiveness of $IL_{17}$ promotion on $M_{c}$	[8,10]	10	pg/mL	ES *
$c_{IL_{1}M_{0}}$	Effectiveness of $IL_{1}$ promotion on $M_{1}$	[8,10]	10	pg/mL	[7]
$c_{IL_{10}M_{0}}$	Effectiveness of $IL_{10}$ promotion on $M_{2}$	[3,5]	5	pg/mL	[7]
$c_{IL_{17}M_{0}}$	Effectiveness of $IL_{17}$ promotion on $M_{1}$	[8,10]	10	pg/mL	ES^*^
$d_{IL_{1}}$	Decay rate of $IL_{1}$	[10,10.5]	10.5	1/day	[7]
$d_{IL_{10}}$	Decay rate of $IL_{10}$	[3,5]	5	1/day	[29]
$d_{IL_{17}}$	Decay rate of $IL_{17}$	[10,10.5]	10.5	1/day	ES *
$d_{R}$	Decay rate of *R*	[10,10.5]	10.5	1/day	ES *
$y_{M_{c}}$	Differentiation rate of *S* to $M_{c}$	[0.7,0.9]	0.9	1/day	[10]
$y_{F}$	Differentiation rate of *S* to *F*	[0.5,0.9]	0.9	1/day	[10]
$m_{M_{c}}$	Effectiveness of $IL_{10}$ promotion on $M_{c}$	[3,5]	5	pg/mL	[10]
$m_{F}$	Effectiveness of $IL_{10}$ promotion on *F*	[3,5]	5	pg/mL	[10]
$\mu_{M_{c}}$	Death rate of $M_{c}$	−	0.3	1/day	[10]
$\mu_{M}$	$M_{0}$, $M_{1}$ and $M_{2}$ emigration rates	[0.1,0.2]	0.2	1/day	[7]
$\mu_{N}$	Rate of neutrophil apoptosis	[0.1,0.3]	0.3	1/day	[30]
$\mu_{S}$	Washout rate of *S*	[1,2]	2	1/day	[10]
$\mu_{T}$	Death rate of γδT	[1,2]	2	1/day	ES *
$\mu_{RX}$	ROS destruction rate of *X*, where	$\left[10^{-4},15\;\times \;10^{-4}\right]$	$15\;\times \;10^{-4}$	1/day	ES *
	$X=M_{c},N,M_{0},M_{1},M_{2},S,T$				
$c_{IL_{17}}$	Half-saturation constant	[95,100]	100	pg/mL	ES *
$c_{RX}$	Half-saturation constant of *X*, where	[95,100]	100	pg/mL	ES *
	$X=M_{c},N,M_{0},M_{1},M_{2},S,T$				

* Estimated parameter values (ESs).

## Data Availability

The original contributions presented in this study are included in this article. Further inquiries can be directed to the corresponding author. The computer codes used for generating the data sets presented in this manuscript are available on GitHub at URL. https://github.com/mehtaplafci/bioengineering-12-00177.git (accessed on 28 January 2025).
